# Differential Expression of Heat Shock Transcription Factors and Heat Shock Proteins after Acute and Chronic Heat Stress in Laying Chickens (*Gallus gallus*)

**DOI:** 10.1371/journal.pone.0102204

**Published:** 2014-07-29

**Authors:** Jingjing Xie, Li Tang, Lin Lu, Liyang Zhang, Lin Xi, Hsiao-Ching Liu, Jack Odle, Xugang Luo

**Affiliations:** 1 Mineral Nutrition Research Division, Institute of Animal Sciences, Chinese Academy of Agricultural Sciences, Beijing, People's Republic of China; 2 Department of Animal Science, North Carolina State University, Raleigh, North Carolina, United States of America; St. Georges University of London, United Kingdom

## Abstract

Heat stress due to high environmental temperature negatively influences animal performances. To better understand the biological impact of heat stress, laying broiler breeder chickens were subjected either to acute (step-wisely increasing temperature from 21 to 35°C within 24 hours) or chronic (32°C for 8 weeks) high temperature exposure. High temperature challenges significantly elevated body temperature of experimental birds (P<0.05). However, oxidation status of lipid and protein and expression of heat shock transcription factors (HSFs) and heat shock proteins (HSPs) 70 and 90 were differently affected by acute and chronic treatment. Tissue-specific responses to thermal challenge were also found among heart, liver and muscle. In the heart, acute heat challenge affected lipid oxidation (P = 0.05) and gene expression of all 4 HSF gene expression was upregulated (P<0.05). During chronic heat treatment, the HSP 70 mRNA level was increased (P<0.05) and HSP 90 mRNA (P<0.05) was decreased. In the liver, oxidation of protein was alleviated during acute heat challenge (P<0.05), however, gene expression HSF2, 3 and 4 and HSP 70 were highly induced (P<0.05). HSP90 expression was increased by chronic thermal treatment (P<0.05). In the muscle, both types of heat stress increased protein oxidation, but HSFs and HSPs gene expression remained unaltered. Only tendencies to increase were observed in HSP 70 (P = 0.052) and 90 (P = 0.054) gene expression after acute heat stress. The differential expressions of HSF and HSP genes in different tissues of laying broiler breeder chickens suggested that anti-heat stress mechanisms might be provoked more profoundly in the heart, by which the muscle was least protected during heat stress. In addition to HSP, HSFs gene expression could be used as a marker during acute heat stress.

## Introduction

High environmental temperature has a harmful impact on an animal's physiology and performance. Different mechanisms are utilized to resist the harmful effects of high temperature depending on duration of exposure. Short-term sub-lethal heat stress (acute heat stress) provokes heat shock response [1]–[3], resulting in rapid initiation of heat shock protein (HSP) synthesis and dramatic changes in gene expression [3]–[6]. In contrast, long-term heat exposure (chronic heat stress) induces larger scale adaptations such as enhancement of endurance by altering thermoregulatory activity (i.e., cardiovascular function). During chronic heat exposure, heat acclimation is also mediated by global molecular responses, including HSP expression [2], [7]. Therefore, HSPs are considered as the cellular thermometer and understanding changes in HSP expression can be useful in evaluating the heat stress response [8].

The HSPs are categorized into several protein families of different molecular weights. The most conserved and best-studied HSP families are HSP 70 and 90 each with several inducible and constitutively expressed members exhibiting different functions. The HSP 70 family binds to newly synthesized polypeptides, prevents aggregation, and assists in folding [9], [10], while HSP 90 interacts with client proteins at a late stage of folding and modifies the configuration of those proteins [11]. At basal and induced levels, the expression of HSP 70 and 90 exhibit spatial and temporal variations [12], [13]. In addition, the duration and severity of heat stress could also influence the expression pattern of HSPs. In ducks, acute heat treatment elevated HSP 90 expression in heart, liver, kidney, and pancreas, whereas chronic heat stress only increased HSP 90 expression in heart and liver [14]. HSP 70 mRNA was induced by increasing environmental temperature to 44°C but not 41°C [15].

Regulation of HSP expression occurs at the transcriptional and translational levels [4], [5], [16]–[18]. Heat shock protein transcription factors (HSFs) act to integrate stress response through transcriptionally activating HSP genes by binding to the heat shock element (HSE) in the upstream promoter region of HSPs [19]. Amongst these factors, HSF1 is the master regulator of the heat shock genes [19]. HSF2 is important for development [6], [16], [19] and also participates in HSF1-mediated HSP expression through formation of a heterocomplex with HSF1 [19]. HSF4 exhibits tissue-specific expression and may function to repress the expression of genes encoding heat shock proteins and molecular chaperones [6], [16], [20]. In avian species, HSF1 and 3 (considered as avian-specific HSF [21], [22]) are the two main HSFs for heat shock response with HSF1 being activated at lower temperatures and the DNA binding activity of HSF3 persisting longer than that of HSF1 [23].

Extensive studies have been carried out to evaluate mechanisms of heat stress in chickens which have mostly focused on acute heat stress in broilers. However, laying broiler breeder chickens are also negatively affected by exposure to high temperature. For example, chronic heat challenge reduced fertility in broiler breeders [24], [25] and produced prolonged decreases in progeny performance [26], [27]. The present study was conducted to determine the effect of both acute and chronic heat stress on body temperature, tissue oxidation status and response of HSP and HSF gene expression in laying broiler breeder chickens.

## Material and Methods

This study was approved by Animal Welfare Committee of Institutes of Animal Sciences, Chinese Academy of Agricultural Sciences (IASCAAS). All experiments were carried out in strict accordance with the recommendations in the Guide for the Care and Use of Animals of the Chinese Academy of Agricultural Sciences and approved by the Animal Welfare Committee of IASCAAS.

Animal husbandry, application of heat stress and sample collection

### Management of broiler breeders

Nineteen-week-old Arbor Acres Female broiler breeders (Huadu Broiler Company; Beijing, China) were raised in two environmentally controlled chicken rooms equipped with cages. The temperature and humidity in both rooms were maintained at 21°C and 40%, respectively. All birds were fed restricted commercial diets (Table 1) in accordance with manufacturer instructions to maintain growth trajectories, and ad libitum access to water was provided. The photoperiodic lighting was programmed with 8-hour light and 16-hour dark per day for the first 3 weeks. Lighting was then gradually increased to 15 hours to promote sexual maturity and maintain growth through 8 wks. After production of the first egg, all females were artificially inseminated with a mixture of semen collected from the same group of roosters twice every week. Males were kept in a chicken house separated from females and raised at 21°C with the humidity of 40%.

**Table 1:** Composition of diets fed to laying broiler breeder chickens.

Ingredients,%	19–24 wk	25 wk-
Corn	67.30	66.63
Soybean meal	19.35	22.38
Wheat bran	7.67	0.00
Lime stone	2.11	7.36
CaHPO_4_	1.76	1.56
Soybean oil	0.71	1.06
NaCl	0.30	0.30
Methionine	0.13	0.18
L-lysine HCl	0.19	0.05
Vitamin and mineral premix^1^	0.48	0.48
Nutrients		
ME, kcal/kg	2800	2800
Crude protein,%^2^	15.8	14.9
Lysine,%	0.78	0.65
Met+ Lys,%	0.67	0.58
Calcium,%^2^	1.09	2.77
Nonphytate phosphorus,%	0.38	0.32

1 Provided per kilogram of diet: VA 15000IU, VD_3_ 4500IU, VE 36IU, VK3 3.9 mg, VB1 4.5 mg, VB_2_ 10.5 mg, VB_6_ 4.5 mg, VB_12_ 0.024 mg, Pantothenic acid calcium 18 mg, Niacin 39 mg, Folic acid 1.5 mg, Biotin 0.18 mg, Choline 1000 mg, Cu (CuSO_4_•5H_2_O) 10 mg, Mn (MnSO_4_•H_2_O) 110 mg, Fe (FeSO_4_•7H_2_O) 60 mg, Zn (ZnSO_4_•7H_2_O) 60 mg, I (KI) 0.35 mg, Se (Na_2_SeO_3_) 0.15 mg.

2 Data based on analyses.

### Acute heat challenge (experiment 1)

**Figure 1:**
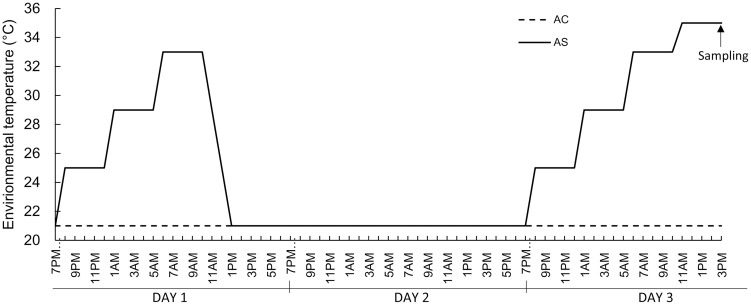
Illustration of thermal application in the acute heat stress treatment. During thermal application to the acute heat stress group (AS, full line), the control group (AC, dotted line) were maintained at 21°C. The increment in the AS chamber temperature started at 7 PM. The environmental temperature was increased by 4°C in 1 hour and held for another 4 hours. After holding at 33°C for 4 hours, the temperature was gradually decreased to 21°C in 3 hours on the first day for adaptation. All 24 birds rested at 21°C for 24 hours and then the procedure was repeated. On the final day (Day 3) of the experiment, instead of returning to 21°C, the AS chamber temperature was continuously increased from 33°C to 35°C and held for 4 hours before sampling.

Twelve laying broiler breeder chickens aged 31 weeks from the two chicken rooms were randomly assigned to one of two treatments with 6 birds per treatment, moved to two environmentally controlled chambers and adapted for 1 week until the acute heat treatment was applied. Feed and water were provided ad libitum. After the adaptation period, the chamber temperature for control birds (acute control, AC) was maintained at 21°C for a duration of three days, while the chamber temperature for acute heat stressed birds (acute stress, AS) was increased step-wise to 33°C on the first day and to 35°C on the third day (Figure 1). Briefly, the increment in the AS chamber temperature started at 19:00 (Day 1) with an increase of 4°C in 1 hour followed by a hold for 4 hours. After the 33°C hold for 4 hours (Day 1), the temperature was gradually decreased to 21°C over a 4 hour period. All 12 birds rested at 21°C for 24 hours (Day 2) and then the procedure was repeated. On the last day (Day 3) of the experiment, instead of returning to 21°C, the AS chamber temperature was increased from 33°C to 35°C and held for 4 hours before sampling. At the end of heat treatment, rectal temperature was recorded and all birds were humanely euthanized by anesthetic overdose for tissue sampling.

### Chronic heat challenge (experiment 2)

Twenty-four laying broiler breeder chickens (29 weeks of age) from the two chicken rooms were randomly assigned to one of two treatments including the chronic control treatment (CC; n = 14) and the chronic heat stress treatment (CS; n = 10). There were 7 replicate cages of two birds per cage for CC and 5 replicate cages of two birds per cage for CS. The CS room was programed for a cyclic room temperature for two weeks to allow the birds to adapt to high environmental temperatures. The cyclic room temperature (25-32-25°C) mimicked the temperature changes of summer day. Briefly, the room temperature was increased to 32°C from 8 AM to 11 AM and returned to 25°C after 4 PM. The temperature was kept at 32°C for the rest of 6 weeks. The CC room temperature was maintained at 21°C for an experimental duration of 8 weeks. Rectal temperature of each bird was measured at 4 PM at the end of each week.

### Sample collection

At the end of each experiment, 6 birds from each treatment were humanely euthanized by anesthetic overdose of Sumianxin II (0.2 ml/kg body weight, Academy of Military Medical Sciences, Changchun, China). Hearts, livers and breast muscles were removed immediately. Parts of these tissues were snap-frozen in liquid nitrogen and then stored at −80°C for the RNA and protein analyses, while the remaining tissue was kept on ice and then transferred to −20°C for subsequent measurement of myocardial malondialdehyde (MDA) activity, protein carbonyl content (PCC) and total superoxide dismutase (TSOD) activity.

### Determination of MDA, PCC and TSOD activity in tissue samples

Tissue samples were homogenized in 10 volumes of physiological saline on ice for 60 sec and then sonicated for 1 min. The homogenates were centrifuged at 1000 g for 15 min at 4°C and supernatants were collected to determine protein content, and MDA and TSOD activity. The total protein content was determined by the Bradford method (Beyotime Institute of Biotechnology, Haimen, China). The level of MDA in the supernatant was determined by the thibabituric acid (TBA) method using a commercial assay (Nanjing Jiancheng Bioengineering Institute, Nanjing, China) and expressed as MDA content per mg protein. Superoxide dismutase 2, mitochondrial (MnSOD) and TSOD activities were measured following the nitrite method described by Li et al [28] and expressed as nitrite units per mg protein.

In order to measure PCC using the spectrophotometric 2,4-dinitrophenylhydrazine (DNPH) assay described by Dalle-Donne et al [29], tissue samples were homogenized in 10 volumes of HEPES (pH 7.2) on ice. Supernatants were separated by 1500 g centrifugation for 15 min at 4°C and 1% streptomycin sulfate was added to minimize the interference from nucleic acids. A solution of 10 mM DNPH in 2 M HCl was added to 100 µl supernatant and kept in the dark for 1 h. Finally, 10% trichloroacetic acid (final concentration) was used for precipitation. After 3 rinses with ethanol/ethyl acetate (1∶1, v/v), the precipitate was resuspended in 6 M guanidine hydrochloride at 37°C for 15 min. The carbonyl contents were measured from the absorbance at 366 nm. The PCC in each tissue sample was expressed as PCC per mg protein.

### Isolation of total RNA, synthesis of cDNA and real time PCR

Total RNA was extracted using TRIzol (Life technologies; Cat # 15596018) following the manufacturers protocol. Briefly, 100 mg tissue was added to 1 ml TRIzol, homogenized and incubated at room temperature for 5 min. A volume of 200 µl chloroform was added to each homogenate and vortexed for 15 sec. The aqueous phase of the sample was separated by 10000 g centrifugation for 15 min at 4°C and transferred carefully to a new vial. The total RNA was precipitated by adding 500 µl of 100% isopropanol and the pellet was obtained by 10000 g centrifugation for 10 min at 4°C. After 2 washes with 70% ethanol, nucleic acid pellets were resuspended in RNase-free water. The concentration of RNA in each sample was determined by NanoDrop Spectrophotomer (ND-1000, Gene company Ltd) and the integrity of total RNA was checked by denatured RNA electrophoresis.

First strand cDNA synthesis was completed using the SuperScript III First-Strand Synthesis system for RT-PCR (Life technologies, Cat # 18080-051) following the manufacturers protocol. Oligo dt was used for cDNA synthesis and 2 µg of total RNA was used. The resulting cDNA was stored at −20°C until use.

Transcriptional expression of 4 HSFs and 2 HSPs was quantified by real-time PCR. Primers (Invitrogen, Beijing) used in the PCR reactions were designed using Primer-BLAST (http://www.ncbi.nlm.nih.gov/). Detailed information on primer sequences is given in Table 2. To determine the amplification efficiency for each primer pair, 5-fold serial dilutions of a cDNA template were analyzed by real-time PCR. The efficiency was calculated as the slope of the linear regression of plotting Ct verse log2-transformed template dilution. PCR reactions (20 µl) contained 1 µl of 5X diluted cDNA, 250 nM of each primer, and PCR buffer master mix from the Power SYBR Green Master Mix (Life Technologies, Cat # 4367659) were carried out using an ABI 7500 Real-Time PCR Detection System (Life Technologies). Each PCR reaction was conducted in triplicate, and the Ct value used in subsequent calculations was the mean of triplicate reactions. The PCR protocol was as follows: denaturation at 95°C for 5 minutes followed by 40 cycles of 95°C for 60 seconds, 60°C for 30 seconds and 72°C for 30 seconds. Dissociation curve analyses were run to ensure specific amplification and verify absence of primer dimers. For each target gene, the cDNA pool of all samples was used as a reference control sample. The geometric mean of internal references, β-actin and GAPDH, were used to normalize the expression of targets genes [30]. The 2^−ΔΔCt^ method was used to calculate mRNA level of each target gene, where the average mean of each age-paired unstressed group was used as the calibrator.

**Table 2:** Information of target genes and primers.

Gene	ID	Primer set (5′-3′)	Product size (bp)	Tm (°C)
β-actin	NM_205518.1	F: 5′-ACCTGAGCGCAAGTACTCTGTCT-3′	95	60
		R: 5′-CATCGTACTCCTGCTTGCTGAT-3′		
GAPDH	NM_204305.1	F: 5′-CTTTGGCATTGTGGAGGGTC-3′	128	60
		R: 5′-ACGCTGGGATGATGTTCTGG-3′		
HSF1	L06098.1	F: 5′-CAGGGAAGCAGTTGGTTCACTACACG-3′	192	60
		R: 5′-CCTTGGGTTTGGGTTGCTCAGTC-3′		
HSF2	NM_001167764.1	F: 5′-CGCTGCTCGCATTCCT-3′	194	60
		R: 5′-TGTGGCCTCACTTGCTTCT-3′		
HSF3	XM_420166.3	F: 5′-TCCACCTCTCCTCTCGGAAG-3′	71	60
		R: 5′-CAACAGGACTGAGGAGCAGG-3′		
HSF4	NM_001172374.1	F: 5′-TGCCAGCCTTCCTAACCAAG-3′	84	60
		R: 5′-TGGTGCCATTCGTACTCCAG-3′		
HSPA2 (Hsp70)	NM_001006685	F: 5′-CGTCAGTGCTGTGGACAAGAGTA-3′	145	60
		R: 5′-CCTATCTCTGTTGGCTTCATCCT-3′		
HSP90AA1 (Hsp90)	NM_001109785.1	F: 5′-GAGTTTGACTGACCCGAGCA-3′	107	60
		R: 5′-TCCCTATGCCGGTATCCACA-3′		

### Statistical analyses

Differences in rectal temperature were determined by Student's t-test at each time points. Data for MDA, PCC, SOD activities, gene expressions were analyzed with one-way analyses of variance (ANOVA) using the GLM procedure in SAS (release 8.0, SAS Institute Inc., Cary, NC), followed by orthogonal contrast analyses to determine the differences in group means of AC and AS, CC and CS, respectively. The cage, or individual bird, served as the experimental unit for all statistical analyses. The data are presented as mean ± SE. Significant differences were considered at the level of P<0.05.

## Results

### Performance of birds during the acute and chronic heat challenges

All birds subjected to thermal treatments displayed heat-dissipating behaviors such as fast panting, wing lifts and extensions. During acute heat stress (experiment 1), when environmental temperature reached 35°C, the average rectal temperature of laying chickens in the acute stress group was increased (P<0.05) to 42.8±0.2°C, compared to 40.6±0.2°C of birds in the AC group.

**Figure 2:**
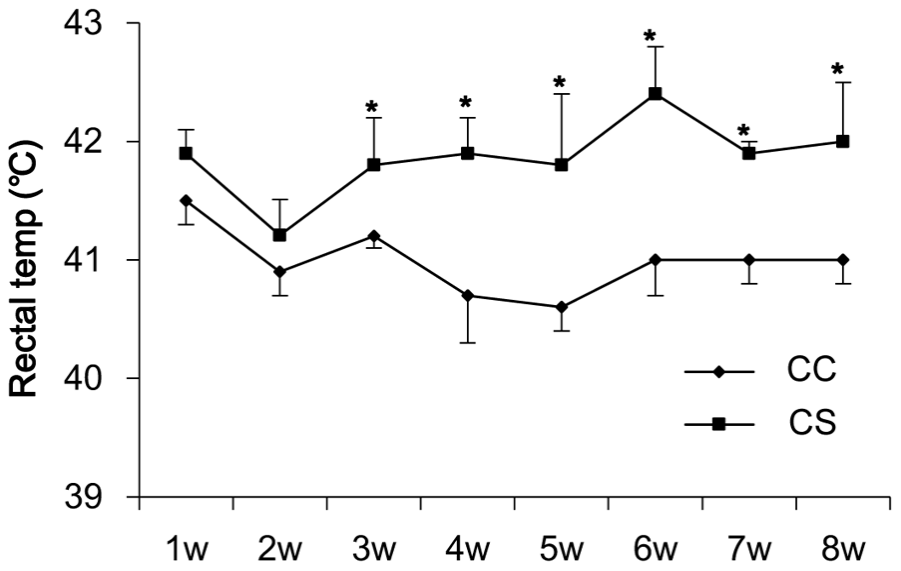
Rectal temperatures of laying broiler breeder chickens in response to the chronic heat challenge at 32°C over 8 weeks. In the first week, the rectal temperature of laying broiler breeder chickens in the chronic heat-stressed group (CS) was not elevated by the cyclic environmental temperature (32-35-32°C) and only a tendency of increase in rectal temperature was observed in the second week. After exposed to the consistent 32°C thermal treatment from the third week, CS birds had a significantly higher average body temperature than those in the chronic control group (CC). Values are expressed as means ± SE of data from 10–12 individual birds. * indicates significant differences (P<0.05) between CS (line with squares) and CC (line with diamonds) at the same time of the trial.

During chronic heat stress (experiment 2; Figure 2), the rectal temperature of birds in CS was not elevated by the cyclic environmental temperature in the first week and only a tendency of increment in rectal temperature was observed in the second week. After exposure to 32°C consistently from the third week on, the CS birds had significantly increased (P<0.05) average rectal temperature of 41.8°C±0.1 compared to 40.9°C±0.1 of control birds. Over the 8-week thermal challenge, heat stressed females had decreased (P<0.05) average feed intake (151.5±0.3 g/d) compared to control birds (162±0.3 g/d) and a reduction (P = 0.06) in average egg production (66.9±4.9%) compared to control birds (81.8±4.9%) during the 8-week experiment.

### Tissue MDA, PCC, TSOD and MnSOD activities after acute and chronic heat stress treatments

**Figure 3:**
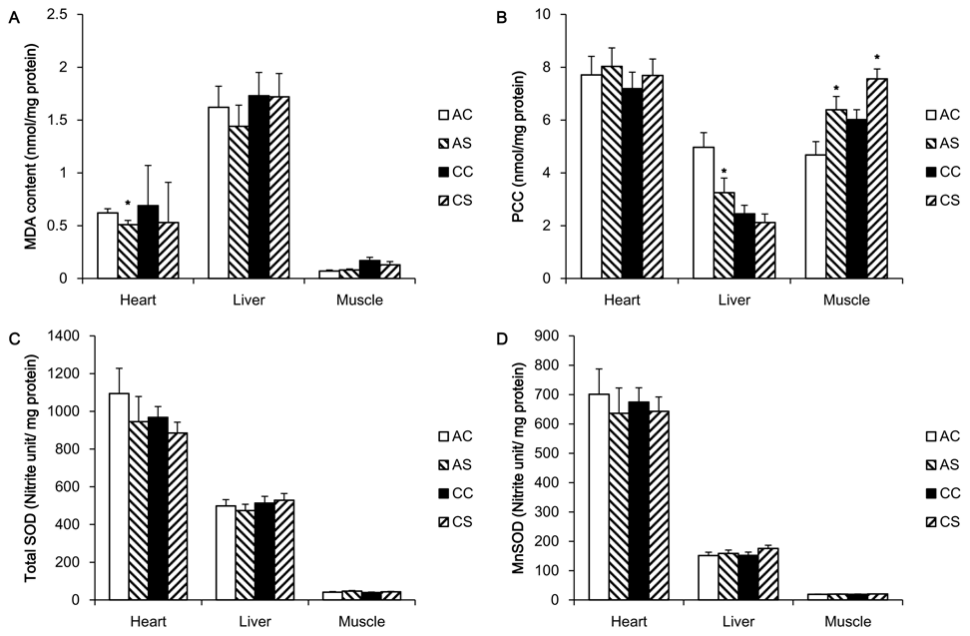
Changes in MDA, PCC and SOD activity after acute and chronic heat challenges. Malondialdehyde (MDA) was used as a biomarker of lipid peroxidation and was determined by the thibabituric acid (TBA) method. Protein carbonyl content (PCC) was employed as a protein oxidation biomarker and was determined using a spectrophotometric 2, 4-dinitrophenylhydrazine (DNPH) assay. Activities of total superoxide dismutase (TSOD) and MnSOD were measured with the nitrite method. Values are expressed means ± SE of data from 5–6 individual tissue samples. * indicates significant differences (P<0.05) between controls and heat-stressed groups. AC, acute control group; AS, acute heat-stressed group; CC, chronic control group; CS, chronic heat-stressed group.

The results of tissue MDA, PCC, TSOD and MnSOD activity from the two experiments are shown in Figure 3A, B, C, and D, respectively. The MDA concentration in the liver was about 2.5 times higher than that in the heart and 14 times higher than muscle. The MDA content in the heart was decreased (P = 0.05) by acute thermal treatment, while hepatic and muscular MDA did not differ between acute heat-stressed and control birds. After an exposure to chronic high temperature at 32°C for 8 weeks, MDA content in all three tissues was not altered.

The PCC in the heart and muscle were comparable, whereas hepatic PCC was about 50% lower than either of these tissues. Both acute and chronic heat stress increased (P<0.05) PCC in muscle. Muscular PCC was increased by 36% and 25% during acute heat stress and chronic heat stress, respectively. Hepatic PCC was decreased (P<0.05) by acute heat stress, but it remained unchanged after chronic heat stress. No change was observed in myocardial PCC between heat stressed and control birds.

The activity of TSOD and MnSOD exhibited a large variation among the three tissues examined, where heart had the highest and muscle had the lowest activity of both dismutases. In all three tissues, there were no significant differences in activity of TSOD or MnSOD after either acute or chronic heat stress treatments.

### Expression of HSFs and HSPs mRNA determined by real-time PCR

**Figure 4:**
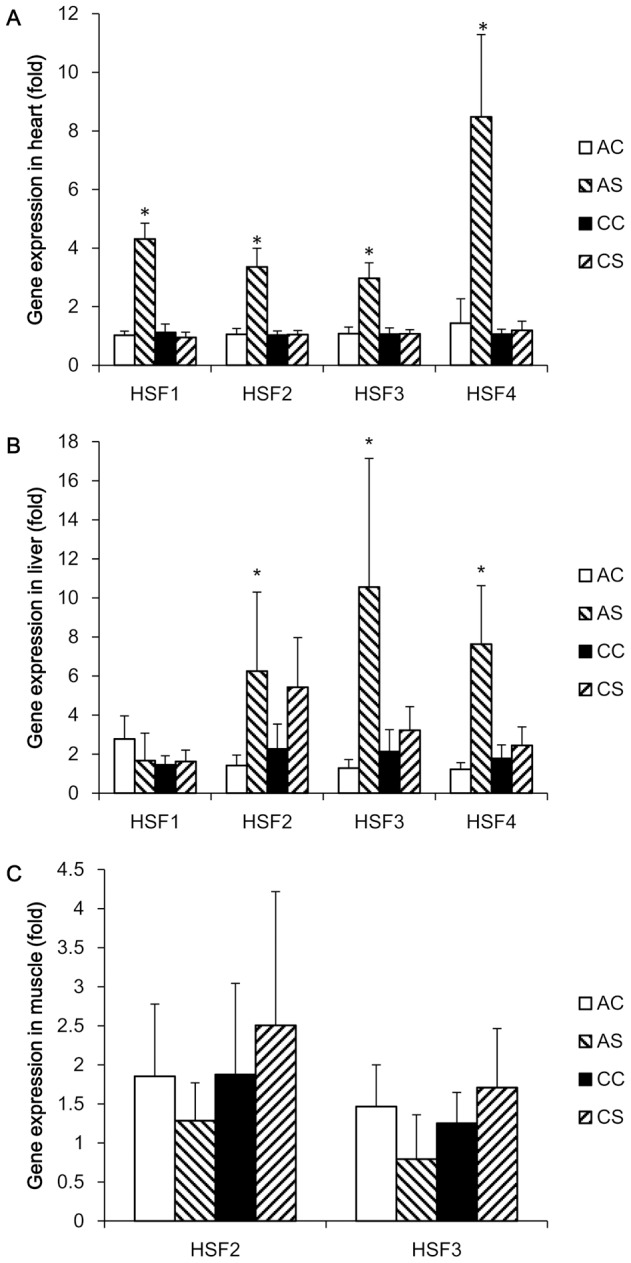
Normalized gene expression of HSF after acute and chronic heat challenges. Expression of heat shock factor (HSF) genes was determined by real time PCR using SYBR green dye and the 2^−ΔΔCt^ method was used to calculate mRNA level of each gene, where the average mean of the unstressed group was used as the calibrator. Each PCR reaction was conducted in triplicate and the geometric mean of internal references, β-actin and GAPDH, was used to normalize the expression of targets genes. Values are expressed as means ± SE of data from 5–6 individual tissue samples. * indicates significant differences (P<0.05) between controls and heat -stressed groups. AC, acute control group; AS, acute heat-stressed group; CC, chronic control group; CS, chronic heat-stressed group.

The results of mRNA expression levels of all 4 HSF genes in the heart, liver, and muscle from the two experiments are shown in Figure 4A, B, and C, respectively. ANOVA analyses showed significant treatment effects in cardiac expressions of HSF1 (F_3,20_ = 36.58, P<0.001), HSF2 (F_3,20_ = 24.83, P<0.001), HSF3 (F_3,20_ = 14.87, P<0.001) and HSF4 (F_3,20_ = 21.75, P<0.001). After acute heat stress, mRNA expression levels of all 4 HSF genes in the heart were significantly increased (HSF1, P<0.001; HSF2, P<0.001; HSF3, P<0.001; HSF4, P<0.001) by 3–8 fold when compared to the AC group. Expression of hepatic HSF2 (P = 0.033), HSF3 (P = 0.04), and HSF4 (P = 0.048) genes were also remarkably elevated by the acute heat stress, whereas no change in hepatic HSF1 expression was observed after the acute heat challenge. In muscle, expression of HSF1 and HSF4 genes were very low regardless of stress status (data not shown), and the expression of HSF2 and HSF3 was not altered by acute heat treatment. No changes in mRNA expression of HSF1, HSF2, HSF3 and HSF4 genes were observed in the heart, liver, or muscle after 8 weeks of chronic heat stress at 32°C compared with its control group (CC).

**Figure 5:**
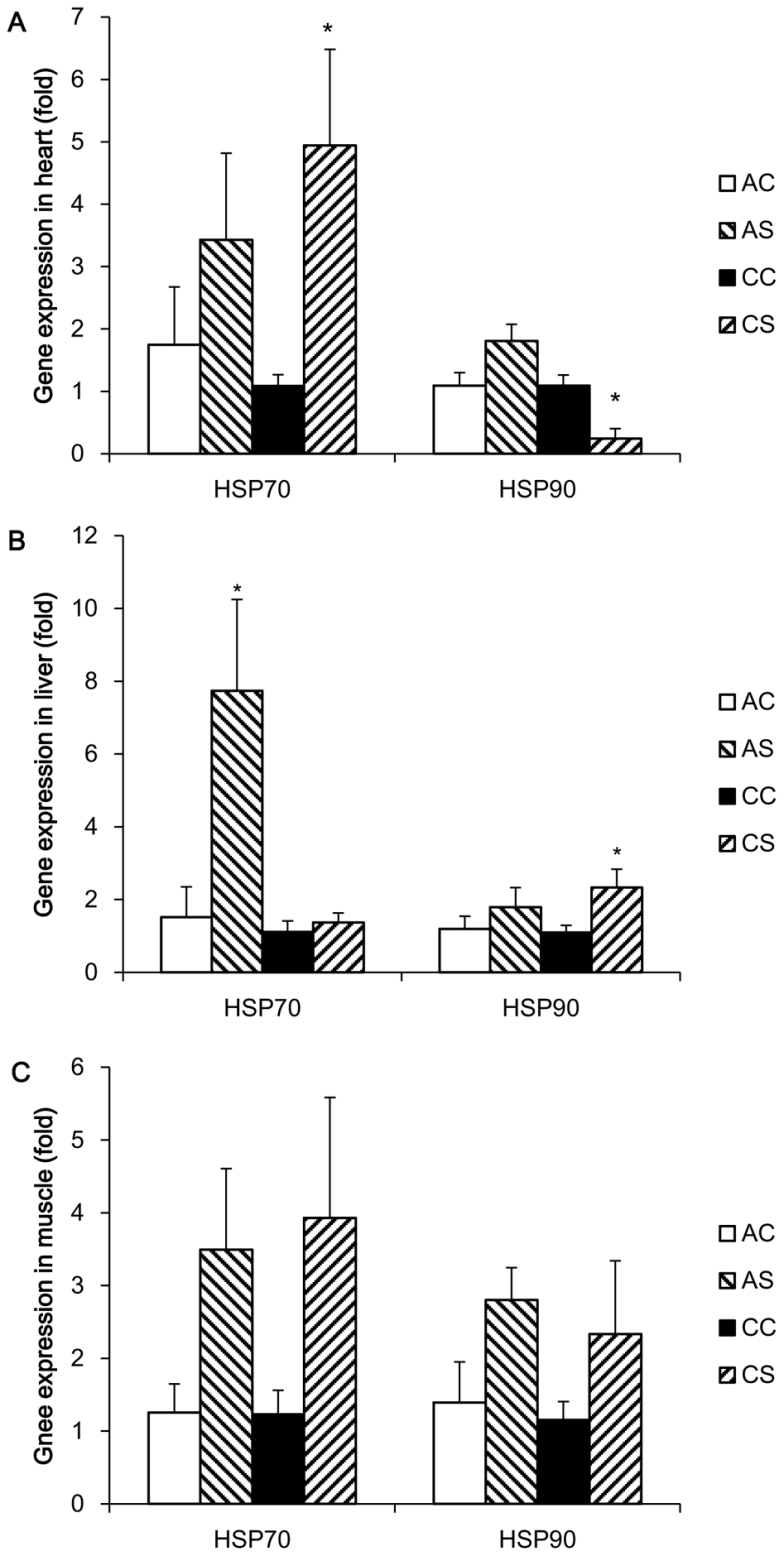
Normalized gene expression of HSP 70 and 90 after acute and chronic heat challenges. Expression of HSP 70 and 90 genes was determined by real time PCR using SYBR green dye and the 2^−ΔΔCt^ method was used to calculate mRNA level of each gene, where the average mean of the unstressed group was used as the calibrator. Each PCR reaction was conducted in triplicate and the geometric mean of internal references, β-actin and GAPDH, was used to normalize the expression of targets genes. Values are expressed as means ± SE of data from 5–6 individual tissue samples. * indicates significant differences (P<0.05) between controls and heat -stressed groups. AC, acute heat control group; AS, acute heat-stressed group; CC, chronic control group; CS, chronic heat-stressed group.

The results of mRNA expression levels of HSP 70 and 90 genes in the heart, liver, and muscle from the two experiments are shown in Figure 5A, B, and C, respectively. Significant treatment effects were demonstrated by ANOVA analyses in HSP 70 and 90 expression in heart (HSP 70, F_3,21_ = 7.088, P = 0.002; HSP 90, F_3,21_ = 9.080, P<0.001), liver (HSP 70, F_3,22_ = 5.720, P = 0.006; HSP 90, F_3,22_ = 4.627, P = 0.013), respectively. In the heart, mRNA expression of HSP 70 was up-regulated (P = 0.049) by chronic heat stress, but not acute heat stress; expression of HSP 90 tended (P = 0.063) to be up-regulated after the acute heat stress, however, it was down-regulated (P<0.001) by the prolonged challenge at 32°C. In the liver, the acute heat stress significantly increased (P = 0.001) HSP 70 gene expression by 7 fold, while no change in its expression was evident after the chronic heat stress treatment. In contrast, HSP 90 expression was up-regulated (P = 0.047) by the chronic heat stress treatment, but not by the acute heat stress treatment. In the muscle, gene expression of HSP 70 (P = 0.052) and 90 (P = 0.054) tended to be increased by acute heat stress; however, expression of these two genes did not differ between the control group and chronic heat stress treatment.

## Discussion

Although the body temperature of laying broiler breeder chickens (reflected by rectal temperature) was significantly increased after acute and chronic thermal stress treatments, the expression of HSF and HSP genes and the oxidation status in examined tissues exhibited remarkable differences between acute and chronic heat-stressed birds. In general, the acute heat challenge at 35°C elevated the rectal temperature of birds by 2°C and had a greater impact on mRNA abundance of HSF and HSP than the chronic heat stress treatment. The response to heat stress also displayed tissue-specific patterns, whereas the heart was the most sensitive and responsive tissue and the muscle was the least responsive to heat challenges.

### Tissue oxidation status and the effect of heat challenges

Heat stress could cause oxidative damage [31]–[33] and lead to accumulation of free radicals and other reactive oxygen species (ROS) [34], [35]. Accumulated ROS activates antioxidant enzymes and damages biological macromolecules such as lipids, proteins and DNA. The oxidation of lipid and proteins can be easily determined by their stable oxidized products. MDA content and PCC are frequently used as biomarkers of lipid peroxidation and protein oxidation, respectively [29]. In the present study, we examined three tissues of great importance in metabolism, heat production and thermal adaption. We found that MDA and PCC varied among tissues. The content of MDA was higher in the liver than in the heart and muscle opposing PCC, which was found in higher levels in heart and muscle. These intra-tissue alterations may arise from differences in lipid and protein deposition in each tissue. In chicken, the liver is the major site of lipogenesis and lipid metabolism, while the heart and muscle are much lower in lipid content. The different content of lipid and protein in the liver, heart and breast muscle may contribute to the variation in basal level of MDA and PCC correspondingly.

Previous studies showed that the production of MDA and PCC was increased in heat-stressed broiler chickens via the overproduction of ROS [35]. In partial agreement, we show herein that protein oxidation in the muscle is induced by both acute and chronic heat stress in laying broiler breeder chickens. Besides heat challenges, age-related oxidation may also affect muscular PCC [36] because PCC was higher in older birds (birds in the chronic heat stress experiment were 2-month older than birds in the acute heat stress experiment). In contrast to muscle, liver PCC was either unaltered or decreased in response to acute or chronic heat challenges. The reduced hepatic PCC during acute heat stress may be associated with decreased feed intake and increased release of protein by the liver [37].

### Differential expression of HSF genes after acute and chronic heat stress

Under stress conditions, especially acute stress, genes related to cell survival are turned on, while less essential genes may be turned off [38]. Our results show that HSF genes display very different profiles after acute and chronic heat stress treatments. HSF genes were observed to be up-regulated after acute heat stress, but did not change after a prolonged stress. Several studies have shown that HSF genes can be induced by certain types of heat stress. In human epidermoid cells, heat treatment elevated the gene expression of HSF 1 and HSP 70 [39]. In plants, gene expression of HSF was altered by heat stress, oxidative stress, and osmotic stress, and the HSF expression changes exhibited a stress-specific and tissue-specific pattern [40]–[42]. The quick response of HSFs, especially during acute heat stress, might be essential for the rapid transcription of HSPs. To date, mechanisms regulating transcription of HSF genes are poorly understood. Xue et al. [43] reported that the C/EBPâ-dependent pathway could be involved in glutamine-induced HSF 1 transcription. According to our data, besides the latency of heat challenge, it seems that temperature might also affect HSF gene transcription. In our experiments, there was a 3°C difference in environmental temperature between acute and chronic heat challenges, which resulted in 1°C difference in average body temperature between birds from the two treatments. The difference in temperature may also contribute to the up-regulation of HSF gene expression. It has been shown that the activation of heat-inducible HSF 1 and 3 require a certain thermal threshold [23]. Whether there is also a temperature threshold to activate gene transcription of HSFs remains unknown.

We also noticed that there were spatial variations in the expression of HSF genes among different tissues even at the basal level. Using similar experimental conditions for real time PCR, the Ct values for muscular HSF1 and HSF4 were above 35 cycles and thus were not reported. Both HSF1 and HSF4 were much less abundant than HSF2 and HSF3 in muscle, and changes in the expression of HSF genes were not evident in the muscle after thermal treatments despite the significant deviations in the heart and liver after acute heat stress. Tissue-specific distribution of HSFs was also observed in humans [20]. HSF1 was found in most tissues, HSF2 was highly present in the heart and liver and HSF4 was observed in the heart, skeletal muscle and pancreas [20]. This tissue-specific expression pattern may be related to the specific roles of each HSF in coping with stress and development [16]. In the liver, HSF1 was not as responsive as the other HSFs in the acute heat treatment. It was recognized that HSF1 is the key integrator for transcription of the classical HSP genes during heat shock for mammals [19]. In chickens, however, HSF3 rather than HSF1, is more potent for thermal-induced expression of HSP genes [16]. To a certain extent, the unchanged HSF 1 expression in the liver after the acute heat stress treatment also supported this idea.

### Differential expression of HSP genes after acute and chronic heat stresses

The activation of HSP 70 and 90 genes during heat challenge has been extensively studied in mammals and birds. In the present study, we found that the patterns of HSP 70 and 90 gene expression in response to different thermal challenges varied among tissues. The abundances of HSP 70 and 90 mRNA were only affected by the chronic heat stress treatment in the heart, while the expression levels of these two genes were up-regulated in the muscle after the acute heat stress treatment. In liver, the acute heat challenge increased HSP 70 mRNA level and the chronic heat stress treatment increased HSP 90 gene expression. Previous studies showed enhanced activation of HSP 70 and 90 genes after acute heat stress. In broiler chickens, acute heat stress induces gene expression of HSP 70 in the liver, lung, heart, kidney, blood vessels [44], [45] and HSP 90 in the heart, liver and kidney [46], [47]. In Shaoxing ducks, HSP 90 gene expression was increased in the heart, liver, kidney, pancreas and pituitary by acute heat stress, and increased HSP 90 expression was limited to the heart, liver, and pituitary after chronic heat stress [14]. The inconsistent results between our study and previous ones may come from model differences and the application of heat challenge. In the study of Shaoxing ducks, the authors found that the HSP 90 mRNA was increased in the heart, liver, and pituitary by the thermal challenge at 35°C, but gene expression in most tissues except pituitary remained unaffected by the treatment at 30°C, suggesting that the temperature of thermal application would be critical to activate HSP gene expression. In the current study, we adopted environmental temperatures of 35°C and 32°C in the acute and chronic thermal challenges, respectively, which were lower than 40°C and 35°C in previous studies on broiler chickens [44]–[47] and ducks [14]. Nonetheless, based upon previous avian studies [14], [44]–[47] and our current study, the heart is the most and muscle the least responsive to heat challenges regarding the activation of HSF and HSP gene expression. Together with the fact that the muscle had the lowest activity of SOD, one of the most important components of tissue anti-oxidative systems, the low responsiveness of HSP 70 and 90 and HSFs in the muscle might contribute to tissue damage by protein oxidation during heat challenges.

Though transcription of HSP genes is known to be potently initiated by binding HSF trimers to the heat shock elements upon stress [4], [18], there were no synchronized changes in transcriptional activation between HSF and HSP genes. There might be other regulatory motifs other than heat shock elements in the promoter regions of HSP genes. In the HSP 70 gene, canonical transcriptional elements TATA, CCAAT, and SP1 binding site were found in the promoter region [48], suggesting the existence of other regulatory mechanisms besides HSFs. Furthermore, based upon the present study, one cannot reach a definitive conclusion given the uncertainty regarding the correlation of HSF mRNA abundance with their DNA binding activities to HSP genes. Accordingly, further analysis of DNA binding activity of HSFs is needed.

## Conclusions

Acute and chronic heat challenges induced different oxidative damage, and altered expression of HSF and HSP genes in the heart, liver and muscle of laying broiler breeder chickens. The heart showed the least oxidative damage, possibly due to having the highest anti-oxidative activity as well as rapid and profound activation of HSF and HSP gene expression. In contrast, muscle displayed the greatest protein oxidation damage, which was paralleled with lowest activity of SOD and refractory responses of HSFs and HSPs. The consistent increased expression of HSF genes suggested its role as a marker for acute heat stress.
